# Frequency-Following Responses for Objective Neurophysiological Monitoring in Pediatric Fetal Alcohol Spectrum Disorder: A Proof of Concept Case Study During Non-Invasive Neuromodulation

**DOI:** 10.3390/children13081116

**Published:** 2026-08-20

**Authors:** Sheila Templado, Raquel Medina-Ramirez, Guillermo Savio, María Teresa Almela, Francisco J. García-Purriños

**Affiliations:** 1 International Doctoral School, Catholic University San Antonio of Murcia (UCAM), 30107 Murcia, Spain; 2 Clínica Templado—Advanced Audiology and Otolaryngology, 30005 Murcia, Spain; 3 Soc-Dig Research Group, University of Las Palmas de Gran Canaria, 35001 Las Palmas de Gran Canaria, Spain; 4 Intelligent Hearing Systems, R&D Department, Miami, FL 33143, USA; 5 Department of Otolaryngology, Virgen de la Arrixaca University Hospital, 30120 El Palmar, Murcia, Spain; 6 Department of Otolaryngology, Los Arcos del Mar Menor University Hospital, 30739 San Javier, Murcia, Spain

**Keywords:** prenatal alcohol exposure, fetal alcohol spectrum disorder, neurodevelopmental disorder, frequency-following response, auditory processing, proof of concept, candidate biomarker, neurophysiological monitoring, child health, non-invasive neuromodulation

## Abstract

**Highlights:**

**What are the main findings?**

- Frequency-Following Responses (FFRs) captured measurable within-subject changes in sustained pitch-encoding metrics over the observation period in a child with Fetal Alcohol Spectrum Disorder (FASD), demonstrating sensitivity as an objective neurophysiological measure.
- Onset response timing, signal-to-noise ratio, and stimulus–response correlation remained stable across recordings, indicating that observed changes were selective to sustained temporal-precision measures rather than to global recording quality.

**What are the implications of the main findings?**

- FFRs may serve as an objective tool for tracking developmental changes in auditory encoding over time in pediatric FASD, motivating further controlled evaluation of FFRs as a candidate tool for longitudinal, non-behavioral monitoring.
- Because this uncontrolled single-case design cannot separate the concurrent neuromodulation from maturation or practice effects, the report is a proof of concept regarding FFR feasibility and not evidence of treatment efficacy.

**Abstract:**

**Background/Objectives:** Fetal Alcohol Spectrum Disorder (FASD) is a preventable neurodevelopmental condition associated with dysfunction of fronto-subcortical networks affecting attention, executive function, and behavioral regulation. Central auditory processing difficulties frequently persist despite preserved peripheral hearing, complicating clinical evaluation when behavioral testing is limited or unreliable, and objective markers that do not depend on behavioral cooperation are therefore needed. This exploratory observational case study investigated whether Frequency-Following Responses (FFRs) can capture measurable within-subject change in speech-evoked neural encoding in a pediatric patient with FASD. **Methods:** FFRs were recorded at two time points bracketing an eight-session period of non-invasive superficial neuromodulation (NESA^®^) in an 8-year-old girl with FASD, using speech syllables (/da/, /ba/, /ga/) presented monaurally at 80 dB SPL. Two-tailed block-level Mann–Whitney U tests with rank-biserial correlations compared peak latencies, sustained pitch-tracking metrics (pitch strength, pitch error), onset stimulus–response correlation, and signal-to-noise ratio. **Results:** Sustained pitch-tracking metrics differed between time points, with increased pitch strength and reduced pitch error, whereas onset peak latencies, onset cross-correlation, and signal-to-noise ratio remained stable. Peak C, marking the transition to the sustained portion of the response, occurred earlier at the post-assessment in all six stimulus–ear combinations. The differences were therefore selective to sustained encoding rather than to onset timing or recording quality. **Conclusions:** FFR-derived pitch-encoding metrics detected measurable within-subject change, providing proof of concept for the feasibility of FFRs as a candidate objective tool for longitudinal monitoring of speech-evoked neural encoding in pediatric FASD. This single-case observation does not evaluate diagnostic accuracy, sensitivity, specificity, or discrimination from typically developing children, and therefore does not establish FFR as a validated diagnostic technique. The findings are hypothesis-generating and motivate controlled longitudinal studies.

## 1. Introduction

Fetal Alcohol Spectrum Disorder (FASD) is one of the leading preventable causes of neurodevelopmental disorders and is characterized by marked clinical heterogeneity, including cognitive, behavioral, attentional, and learning impairments. Prenatal alcohol exposure induces persistent structural and functional changes in the central nervous system, particularly affecting fronto-subcortical networks involved in executive functions, inhibitory control, emotional regulation, and attention. These alterations may occur even in the absence of evident dysmorphic features, contributing to delayed diagnosis and underdetection of the condition [1,2,3].

Within the neuropsychological profile of FASD, increasing attention has been directed toward difficulties in sensory information processing, particularly auditory processing. Many children with FASD exhibit persistent problems in speech perception—especially in noisy environments—along with deficits in temporal discrimination, sustained auditory attention, and auditory–linguistic integration. This pattern is consistent with auditory processing disorder (APD), characterized by listening difficulties despite preserved peripheral hearing. In this context, APD should be understood not as an isolated auditory alteration, but as the functional expression of broader disruptions in neural synchronization and top-down control mechanisms [4,5].

Objective assessment of auditory processing in pediatric populations with FASD presents significant methodological challenges, as traditional behavioral tests may be limited by age, attentional capacity, cognitive difficulties, or language, reducing their clinical reliability. In this context, electrophysiological techniques provide a valuable alternative. Among these, the frequency-following response (FFR) provides an objective electrophysiological index of neural encoding of temporal and spectral sound features, offering a sensitive measure of neural synchrony and temporal precision at brainstem-dominated levels of processing. FFR has demonstrated sensitivity to neurodevelopmental alterations and auditory processing deficits, supporting its use as a candidate biomarker for longitudinal assessment. Its clinical applicability has been examined across pediatric populations, where altered FFR measures have been documented in auditory processing disorder, developmental language disorder, and reading difficulty [6,7,8,9,10]. Normative characterization of the FFR in typically developing school-aged children supports its use as an objective reference against which individual profiles can be compared [11].

In a related case-control study, our group recently reported that individuals with FASD showed reduced pitch-tracking consistency, lower stimulus–response correlation, and prolonged response latencies in speech-evoked FFRs compared with typically developing controls, establishing the feasibility of the FFR as a candidate auditory biomarker in this population [12].

That work established the group-level foundation for the present line of research: it characterized how speech-evoked encoding differs between individuals with FASD and typically developing controls at a single time point, and identified longitudinal, within-subject monitoring as the necessary next step. The present report takes that step. Having established that FFR measures are altered at the group level in FASD, the question that follows is whether the same measures are sensitive enough to track change within a single individual over time—a property that cross-sectional comparison cannot address and that any monitoring application requires.

The acquisition parameters, analysis pipeline, and outcome metrics are common to both studies. The protocol was established for the reference cohort and has since been adopted as the standard neurophysiological evaluation for auditory processing referrals in our clinical practice, where it is applied in extended form: whereas the group-level study elicited responses to the syllable /da/ alone, the clinical protocol uses three syllables (/da/, /ba/, /ga/), so that encoding can be characterized across stimuli differing in place of articulation rather than at a single point.

The participant described in this report was assessed in 2025 and did not form part of the earlier cohort, which was recruited between 2023 and 2024; the two datasets are independent, and the shared core protocol allows individual cases to be interpreted against the reference data, with /da/ common to both, and compared with future cases assessed under the same procedure.

Although the FFR has traditionally been interpreted as a predominantly subcortical response, converging magnetoencephalographic evidence indicates that it is in fact a multi-site response, with concurrent contributions from cortical (including auditory cortex) and subcortical generators, rather than a signal of purely brainstem origin that is only modulated top-down [13,14,15].

Consistent with this distributed origin, growing evidence indicates that its functional expression is further shaped by the state of higher-order cortical networks. In particular, the prefrontal cortex plays a central role in top-down auditory modulation, influencing processes such as auditory attention, detection of relevant acoustic changes, and optimization of sensory encoding. Neurophysiological and neuroimaging studies have demonstrated the involvement of prefrontal regions in pre-attentional detection and in the modulation of both cortical and subcortical auditory responses [16].

From this perspective, prefrontal dysfunction—common in FASD—may indirectly contribute to suboptimal subcortical auditory encoding, affecting temporal consistency and precise tracking of speech features. Non-invasive neuromodulation has emerged as one approach explored in neurodevelopmental disorders with the aim of modulating cortical excitability and promoting functional reorganization within altered neural networks, with the prefrontal cortex representing a clinically relevant target due to its role in regulating arousal, attention, executive control, and sensory modulation [17].

Non-invasive electrical neuromodulation techniques, including transcranial direct current stimulation (tDCS), have been shown to modulate cortical excitability and functional networks [18]. Within this framework, low-intensity superficial pulsed microcurrent neuromodulation has been proposed as a safe adjunct in pediatric populations, with reported effects on autonomic regulation and higher-order cortical function [19,20,21,22]. This body of preliminary evidence is cited here strictly as clinical background motivating the context in which the present electrophysiological observation was made; it is not offered as established support for a therapeutic effect.

Beyond its teratogenic, non-genetic origin, FASD shares with many complex and rare pediatric neurodevelopmental conditions a core diagnostic challenge: marked phenotypic heterogeneity, frequent delay in identification, and limited reliability of behavioral testing in early childhood. Within this broader landscape of pediatric conditions in which diagnosis and monitoring are complicated by variable presentation and uncooperative or unreliable behavioral response, the development and validation of objective, non-invasive physiological biomarkers capable of tracking neural function independently of behavioral cooperation is of clinical relevance across etiologies. FFRs, as a scalable and objective electrophysiological measure, are well positioned to contribute to this broader effort in pediatric diagnostic neurophysiology.

Building on this group-level evidence of altered neural speech encoding in FASD, no study to date has examined FFR-based indices of speech-evoked neural encoding as a longitudinal, within-subject monitoring tool in this population. Given the limitations of behavioral assessment in FASD and the putative involvement of fronto-subcortical networks in auditory processing deficits, FFR offers a well suited, physiological approach providing an objective window into speech-evoked neural encoding over time—one that may be informative in a population where standard behavioral markers are often unreliable.

The present study reports an exploratory, observational case in which FFRs were recorded in a child with FASD at two time points, bracketing an eight-session period of non-invasive neuromodulation (NESA^®^) that was proposed following the auditory processing diagnosis as an experimental, non-pharmacological option. The primary aim of this report is to assess the feasibility and sensitivity of FFR-derived measures as a candidate objective neurophysiological marker for tracking speech-evoked neural encoding over time in a single pediatric case. No claim of diagnostic validity is made: the present design does not assess diagnostic accuracy or group discrimination. The report is intended to illustrate the broader need for objective, non-behavioral physiological measures in the evaluation of heterogeneous neurodevelopmental conditions such as FASD.

## 2. Materials and Methods

### 2.1. Participant

The participant was an 8-year-old female with a clinical diagnosis of fetal alcohol spectrum disorder (FASD), established during follow-up for persistent neurodevelopmental difficulties involving attention, learning, and information processing. The facial phenotype characteristic of fetal alcohol syndrome was not present, consistent with a presentation within the FASD spectrum without the full facial features. The FASD diagnosis had been established previously by the specialist unit of the regional reference hospital, following clinical evaluation that included assessment by the clinical psychology service; prenatal alcohol exposure was documented in the referring clinical record. The diagnosis was not independently re-established by the present authors, and no formal diagnostic coding system (e.g., the 4–Digit Diagnostic Code or updated clinical FASD diagnostic criteria were available in the referring report. She was subsequently referred for specialized auditory processing evaluation on the basis of behavioral markers of impaired auditory attention and auditory memory, together with reduced academic performance not attributable to peripheral hearing impairment.

### 2.2. Audiological and Neurodevelopmental Assessment

Peripheral hearing was normal, with preserved cochlear function and no relevant otologic history. The functional profile was consistent with auditory processing disorder (APD), marked by deficits in auditory attention and speech perception in adverse listening conditions. These findings were interpreted within the broader fronto-subcortical neurofunctional involvement associated with FASD. Detailed procedures are provided in the Supplementary Materials.

### 2.3. Observational Design and Timeline

Baseline (pre) recordings were obtained approximately eight weeks before the first neuromodulation session. This gap was determined by clinical scheduling constraints rather than by design: the baseline auditory processing evaluation was performed in the closing weeks of the academic year, and the neuromodulation sessions were subsequently scheduled according to clinical availability during the following school holiday period. The eight sessions were delivered twice weekly over four weeks. Post recordings were acquired immediately after completion of the eighth session, during the same clinical visit. The two assessments were therefore approximately twelve weeks apart. No additional intervention occurred between the final neuromodulation session and the post-assessment.

Throughout the observation period the participant received no concurrent therapeutic interventions of any kind—including speech and language therapy, occupational therapy, auditory training, or pharmacological treatment. No additional school-based support program was in place: the observation period spanned the transition from the final weeks of the academic year to the school holiday period, and the neuromodulation sessions themselves fell entirely within the holiday period. The non-invasive neuromodulation described in Section 2.6 was therefore the only structured intervention administered between the two assessment time points.

### 2.4. FFR Acquisition and Signal Processing

Frequency-following responses (FFRs) were recorded using the SmartEP^®^ v. 2.93.10 software and Duet^®^ system (Intelligent Hearing Systems, Miami, FL, USA). Four surface electrodes were positioned according to the International 10–20 EEG system: active at Cz, reference at the ipsilateral mastoid (M1 or M2), and ground at Fpz. Electrode impedances were maintained below 2 kΩ at all recording sites. Although the system allows two-channel acquisition, recordings were performed separately for each ear, with a single recording channel active per run corresponding to the stimulated ear. Speech-evoked FFRs were elicited using the syllables /da/, /ba/, and /ga/, generated within the SmartEP^®^ Advanced Auditory Research Module, each with a fundamental frequency (F0) of 97.66 Hz. Stimulus duration was 160 ms, and the presentation rate was 4.35/s, corresponding to an inter-stimulus interval of 229.89 ms. Stimuli were delivered monaurally at 80 dB SPL via UltraShield ER-3 insert earphones (Etymotic Research, Elk Grove Village, IL, USA), calibrated according to the manufacturer’s standard clinical procedure. Stimulus polarity was set to alternating to minimize stimulus artifact and cochlear microphonic contributions. Recordings were conducted inside a sound-attenuated and electrically shielded booth. Signals were amplified (gain = 100,000), digitized at a sampling interval of 75 µs (≈13.33 kHz), and recorded using an online band-pass filter of 30–3000 Hz. A 40-ms pre-stimulus baseline was applied, resulting in a total acquisition window of approximately 230 ms. Online artifact rejection was applied at ±31 µV within the 0–230 ms recording window relative to buffer onset. Each stimulus condition consisted of three independent recording blocks of 1024 sweeps each (3072 sweeps per stimulus, per ear, and per session). Accepted sweeps were averaged to obtain one waveform per block; block-level averaged waveforms were retained separately for quantitative and statistical analyses.

Recordings at both time points were obtained with the same Duet^®^ system and SmartEP^®^ software, using identical calibration, electrode montage, stimulus parameters, and acquisition settings, within the same sound-attenuated and electrically shielded booth, and were performed by the same examiner. Recording conditions were standardized across both assessments: the participant was positioned supine on an examination couch in a relaxed state, and stimulus presentation was initiated only once the ongoing EEG had stabilized, so that acquisition began from an equivalent baseline state at both time points. The participant tolerated the protocol well and remained in a relaxed, still state throughout acquisition, without myogenic contamination of the recording and without any need for sedation. Sleep staging was not performed, and no formal classification of arousal state was therefore applied. Recording quality was monitored online during each session via electrode impedance, artifact rejection rate, and visual inspection of the ongoing EEG, which remained of high quality across both assessments.

Offline digital band-pass filtering (70–2000 Hz) was applied uniformly to all block-level waveforms prior to analysis. The 70 Hz high-pass cutoff was selected to attenuate slow baseline drifts and low-frequency components not phase-locked to the stimulus. The 2000 Hz low-pass cutoff was applied to reduce high-frequency noise while preserving the fundamental frequency (F0 = 97.66 Hz) and its relevant harmonic components within the speech-evoked response.

### 2.5. Spectral Analysis and Pitch Tracking

Spectral and pitch-tracking analyses were conducted using the Brainstem Toolbox (Auditory Neuroscience Laboratory, Northwestern University, Evanston, IL, USA; pitch-tracking module, October 2011 release), following established procedures for speech-evoked auditory brainstem responses [8]. For pitch tracking, an autocorrelation-based approach was applied from 0 to 150 ms relative to stimulus onset using a 40-ms sliding analysis window with a 1-ms step size. The expected neural lag parameter was set to 10 ms, corresponding to the period of the stimulus fundamental frequency (F0 = 97.66 Hz). The target frequency range for neural response tracking was set to 0–120 Hz, and the stimulus target range to 80–120 Hz. Pitch strength was defined as the magnitude of the normalized autocorrelation peak at the lag corresponding to the expected F0 period (approximately 10.24 ms). The neural pitch estimate was derived from the lag yielding the maximum autocorrelation coefficient within the predefined search range and converted to frequency (Hz); pitch error was calculated as the absolute difference between the stimulus F0 (97.66 Hz) and the extracted neural pitch estimate, following established FFR analysis procedures [8,23]. For statistical comparison, block-level estimates of pitch strength and pitch error were extracted for each stimulus and ear.

Four metrics were selected to cover complementary properties of the response and to allow specific effects to be distinguished from non-specific ones. Pitch strength and pitch error together characterize the sustained portion of the FFR: the former indexes the consistency with which the periodicity of the stimulus is represented, and the latter the accuracy of that representation relative to the stimulus fundamental frequency. These are related but separable, since a response may track F0 consistently at a systematically displaced frequency, or accurately but with low phase consistency, so reporting both provides a fuller description than either alone. Onset stimulus–response cross-correlation (3.98–9 ms) and signal-to-noise ratio were included as control measures rather than as outcomes of interest: the former indexes the fidelity of the transient onset response, which arises from processes distinct from sustained periodicity encoding, and the latter indexes overall recording quality. Because differences in arousal, electrode impedance, or acquisition conditions would be expected to affect these two measures alongside the sustained metrics, their stability provides an internal check on whether any difference observed in the sustained metrics is specific rather than attributable to global recording factors. All four are standard measures in the speech-evoked FFR literature and are computed by the analysis toolbox used here [8,23].

### 2.6. Concurrent Clinical Intervention (Non-Invasive Neuromodulation)

Following the auditory processing disorder diagnosis, non-invasive neuromodulation was proposed as an experimental, non-pharmacological option and was delivered using a superficial electrical neuromodulation device (NESA^®^, NESA World, San Sebastián—Donostia, Spain). Although electrical stimulation is applied at distal surface sites, the manufacturer’s rationale for this device is based on systemic modulation of large neuronal populations through peripheral afferent pathways and autonomic regulatory mechanisms. Stimulation was applied via 24 distal surface electrodes (six per limb), integrated into glove and anklet interfaces, with the electrical circuit completed by a directional electrode whose position determined the functional orientation of the stimulation axis: a central posterior axis (directional electrode at C7) or an anterior cranial orientation axis (directional electrode at Fpz, according to the 10–20 EEG system).

Low-frequency pulsed microcurrents within the operational range of the device (1.14–14.28 Hz; 0.1–1.0 mA; ±3 V) were delivered across eight 60-min sessions administered twice weekly, producing no perceived sensory experience and no observable motor activation. All externally configurable parameters (frequency range, voltage, session duration, electrode positioning, and program selection) are specified below to ensure procedural replicability; the internal modulation algorithm is proprietary and not publicly disclosed, which is acknowledged as a methodological limitation affecting mechanistic interpretation (see Section 4.5). Stimulation followed a fixed program sequence: Session 1 comprised Program 1 (30 min, directional electrode at C7) followed by Program 8 (30 min, at Fpz); Sessions 2–4 comprised Program 1 (15 min, at C7) followed by Program 8 (45 min, at Fpz); Sessions 5–6 comprised Program 8 (15 min, directional electrode at C1–C2) followed by Program 8 (45 min, at Fpz); and Sessions 7–8 comprised Program 8 (60 min, at Fpz) only. Program 1 is described by the manufacturer as a global modulator of the autonomic nervous system, applying sequential changes in stimulation timing and frequency across the 24 electrodes via polarity-shifting impulses; positioning the directional electrode at C7 establishes a central posterior axis and was used as an initial physiological adaptation phase. Program 8 applies sequential microcurrent patterns with symmetrical polarities across positive and negative channels within a low-frequency range (~7.69 Hz); with the directional electrode at Fpz, the circuit establishes an anterior cranial orientation axis that, according to the manufacturer’s technical protocol, is intended to functionally direct modulation toward anterior regions, including prefrontal areas. Sessions were conducted with the participant in a seated resting position; the intervention was entirely passive, and no adverse effects were reported. As detailed in Section 2.3, no concurrent therapeutic or pharmacological interventions were administered during the observation period.

### 2.7. Statistical Analysis

Given the single-case design, statistical comparisons were conducted at the block level. For each stimulus (/da/, /ba/, /ga/), three independent recording blocks were obtained per condition and ear, yielding nine block-level observations per condition per ear (*n* = 9). When both ears were analyzed together, 18 observations per condition were included (*n* = 18). Blocks represent averages of non-overlapping sets of sweeps acquired sequentially within each session. Because pre- and post-recordings were obtained in separate sessions, block identity across sessions was not assumed to reflect matched physiological events.

Normality assumptions were not met; therefore, comparisons between time points were performed using independent-samples Mann–Whitney U tests, separately for each ear and for the combined-ear analysis. All tests were two-tailed, and no directional hypothesis was specified a priori, consistent with the exploratory nature of this report. Effect sizes were quantified using the rank-biserial correlation, reported alongside each comparison in Table 1; effect magnitude is reported alongside statistical significance and is given equal weight in the interpretation of the results.

Twelve comparisons were performed across four metrics, two single-ear analyses, and one combined-ear analysis. No formal correction for multiple testing was applied, and *p*-values are therefore reported uncorrected; they are interpreted descriptively rather than as confirmatory evidence, consistent with the exploratory purpose of this report. Two considerations are relevant to their interpretation. First, the single-ear analyses are not independent of the combined-ear analysis, since the latter pools the same observations, so the twelve tests do not represent twelve independent opportunities for a false positive. Second, the observed pattern is not one of isolated significant findings distributed at random: statistically significant differences were confined to the two sustained pitch-encoding metrics and were consistent in direction across ears, whereas the two control metrics (SNR and onset cross-correlation) showed no differences and small effect sizes in every comparison. While replication remains necessary, this internal consistency is not the pattern expected if the observed differences were driven by multiple testing alone. Analyses were conducted in jamovi (version 2.0.0; the jamovi project, Sydney, Australia).

Block-level observations are repeated acquisitions from the same participant and do not constitute independent biological observations; the biological sample size here is one, and no population-level inference is drawn. The block-level analysis quantifies the magnitude of within-subject change relative to the measurement variability of the technique itself—the relevant quantity when assessing whether an electrophysiological measure is sensitive enough to register change in a single patient. This constraint is structural to single-case designs, and the single-case literature developed in direct response to it: non-overlap indices such as Tau-U exist precisely because standard independence assumptions are routinely violated in these data [24]. In its two-phase form without a trend component, Tau-U is mathematically equivalent to the Mann–Whitney U test and returns identical *p*-values [24,25]; its added value lies in adjusting for within-phase trend, which requires enough sequential observations per phase to estimate trend reliably. With three blocks per phase for each stimulus and ear, trend estimation would be unstable, making Mann–Whitney U the appropriate analytic choice here rather than a weaker substitute. Non-independence across the three blocks acquired within a given stimulus and session remains, and it is for this reason that effect magnitudes and the internal consistency of the response pattern carry the interpretive weight in this report.

## 3. Results

### 3.1. Sustained and Onset FFR Metrics

Exploratory block-level statistical comparisons are summarized in Table 1.

Pitch strength values were higher in the post recordings when both ears were analyzed together (Mann–Whitney *U* = 67.00, *p* = 0.003, *r* = 0.59). Median pitch strength increased from 0.39 at the pre-assessment to 0.71 at the post-assessment. In exploratory ear-specific analyses, the difference reached statistical significance in the left ear (*U* = 13.00, *p* = 0.017, *r* = 0.68), while the right ear showed an effect of comparable magnitude that did not reach significance at the conventional threshold (*U* = 22.00, *p* = 0.112, *r* = 0.46), consistent with the reduced number of observations available in single-ear comparisons (*n* = 9 per condition).

**Figure 1:**
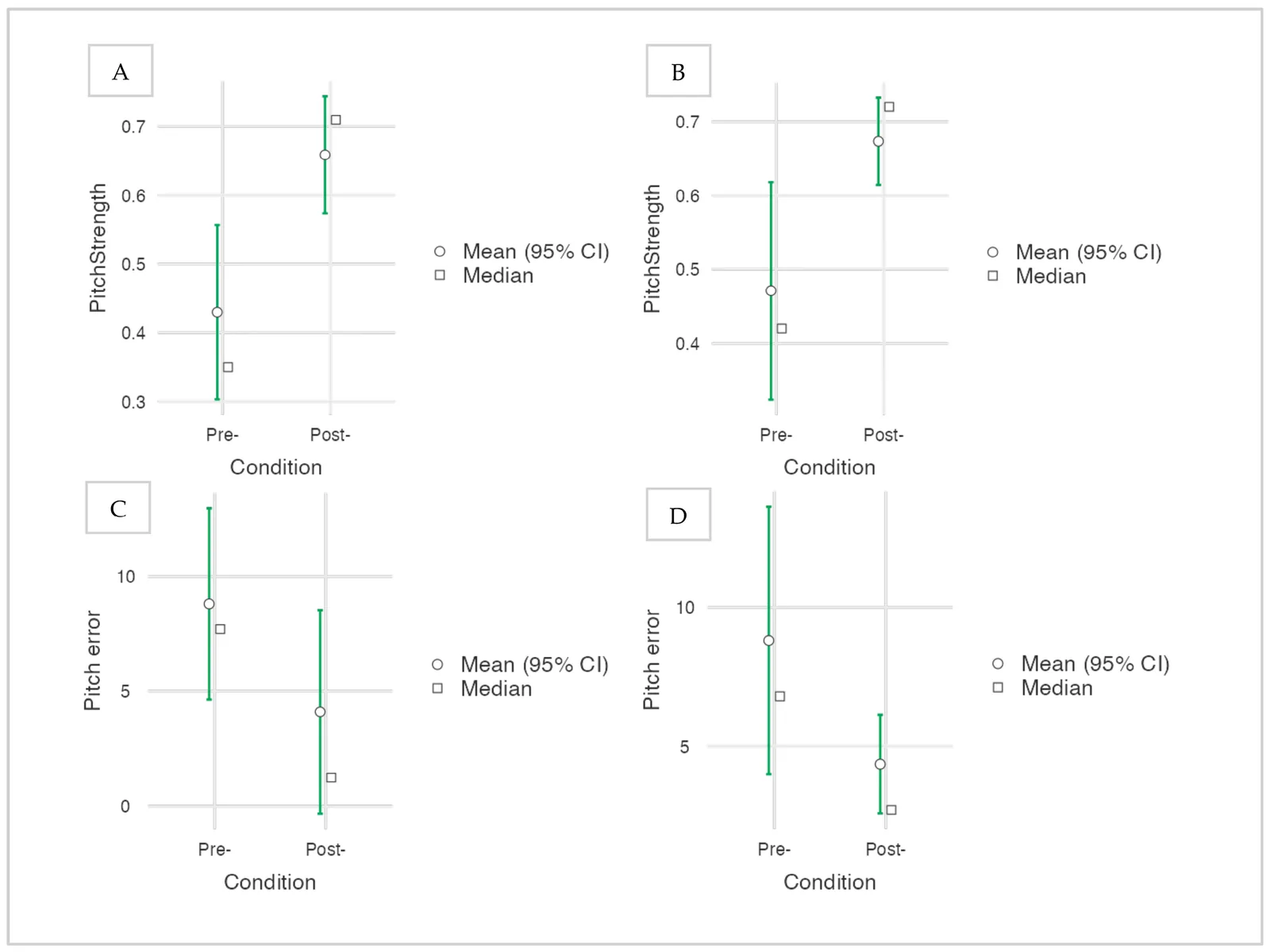
Block-level changes in sustained FFR pitch-encoding metrics between the two assessment time points. (**A**) Pitch strength—left ear; (**B**) Pitch strength—right ear; (**C**) Pitch error—left ear; (**D**) Pitch error—right ear. Metrics were derived from the sustained portion of the FFR (0–150 ms) using an autocorrelation-based pitch-tracking method. Circles represent mean values (95% CI); squares represent median block-level values (*n* = 9 per condition per ear).

Pitch error demonstrated a complementary pattern. In the combined-ear analysis, pitch error values were lower in the post recordings (*U* = 87.50, *p* = 0.019, *r* = −0.46), with median values decreasing from 7.25 to 2.54. Exploratory ear-specific analyses showed a reduction of large magnitude in the left ear that reached trend level (*U* = 19.50, *p* = 0.070, *r* = −0.52) and a smaller, non-significant reduction in the right ear (*U* = 27.00, *p* = 0.251, *r* = −0.33). Pitch strength and pitch error changes across the two assessment time points are illustrated in Figure 1.

Recording quality remained stable across sessions, as reflected by comparable signal-to-noise ratio (SNR) and onset cross-correlation values. SNR did not differ between time points either in the combined-ear analysis (*U* = 146.00, *p* = 0.624, *r* = −0.10) or in ear-specific analyses (right ear: *U* = 38.50, *p* = 0.894, *r* = 0.05; left ear: *U* = 30.00, *p* = 0.377, *r* = −0.26). Similarly, cross-correlation values within the onset window (3.98–9 ms) showed no differences between sessions for the combined-ear analysis (*U* = 152.00, *p* = 0.764, *r* = −0.06), the right ear (*U* = 40.50, *p* = 1.000, *r* = 0.00), or the left ear (*U* = 35.00, *p* = 0.659, *r* = −0.14). No consistent changes were observed in onset latency measures at the descriptive level.

Overall, the differences observed were confined to the two sustained pitch-encoding metrics, where effect sizes were moderate to large in every comparison (|*r*| = 0.33–0.68) and consistent in direction across both ears. The two control metrics showed small effect sizes throughout (|*r*| ≤ 0.26), with no differences between time points (Table 1).

### 3.2. Temporal Peak Latencies and Waveform Morphology

Peak latencies were identified on the waveform obtained by averaging all three blocks per stimulus and ear (3072 sweeps), since reliable peak identification required the improved signal-to-noise ratio of the fully averaged response; they are therefore reported descriptively and were not included in the block-level comparisons of Table 1. Onset components were stable across time points: peak V varied by less than 0.7 ms in every stimulus–ear combination, and peak A showed no consistent direction of change (Supplementary Table S1). Peak C, which marks the transition between the onset response and the sustained periodic portion of the FFR, occurred earlier at the post-assessment in all six stimulus–ear combinations, by 4.06–6.23 ms (mean 5.04 ms). Peaks D, E, and F showed no consistent shift, while peak O occurred earlier at the post-assessment in five of six combinations.

**Figure 2:**
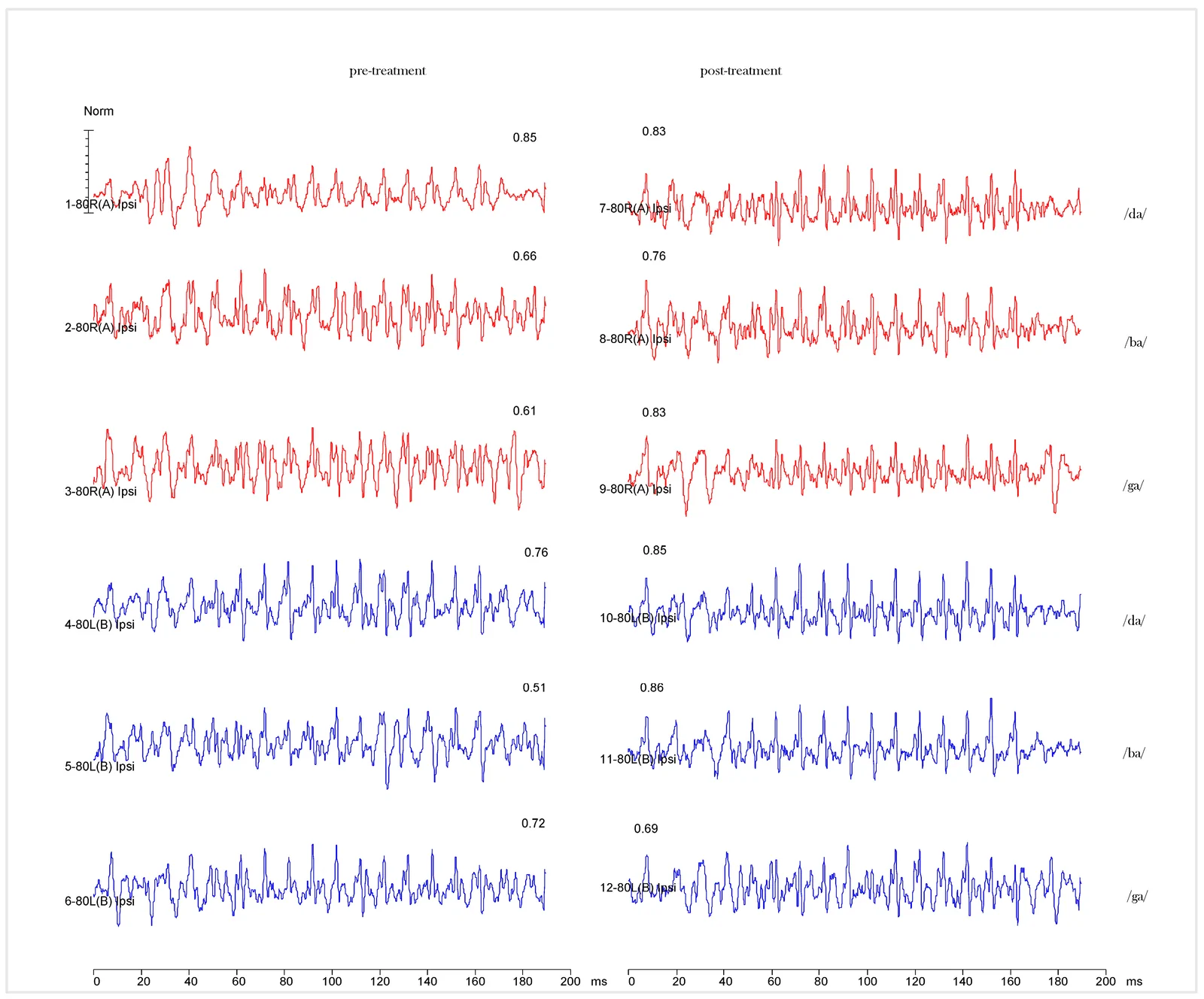
Representative speech-evoked FFR waveforms at the two assessment time points. Grand-averaged waveforms elicited by /da/, /ba/, and /ga/ are shown for the right ear (red) and left ear (blue), pre (left column) and post (right column). Each waveform is the average of three sequential recording blocks (3 × 1024 sweeps; 3072 sweeps total). Numbers displayed above each waveform indicate the pitch strength value computed for that waveform using the Brainstem Toolbox (autocorrelation-based method). Block-level statistical comparisons of sustained pitch-encoding metrics are reported in Figure 1 and Table 1.

Representative FFR waveforms are shown in Figure 2. Stimulus-specific pitch-tracking correlation values for each syllable and ear are reported in Supplementary Table S2, and temporal pitch-tracking trajectories are illustrated in Supplementary Figure S1. Time–frequency representations derived from running FFT analysis are provided in Supplementary Figure S2.

## 4. Discussion

### 4.1. Main Findings

This proof of concept case study describes measurable within-subject differences in frequency-following response (FFR) measures between two assessment time points bracketing an eight-session period of non-invasive superficial neuromodulation with anterior cranial orientation in a pediatric patient with FASD. Sustained pitch-encoding metrics differed significantly between time points, including increased pitch strength and reduced pitch error, while onset peak latencies, signal-to-noise ratio, and onset cross-correlation (3.98–9 ms) showed no systematic shifts. This pattern suggests change in sustained temporal precision of auditory neural encoding rather than reorganization of response timing, and is consistent with change in functional encoding stability rather than structural alteration of transmission along the auditory pathway [26].

The level at which these differences arise requires specification, since it constrains how the pattern can be interpreted. The scalp-recorded FFR reflects a distributed set of generators comprising the auditory nerve, the brainstem and inferior colliculus, and bilateral primary auditory cortex, whose contributions decline steeply along the neuroaxis in parallel with the roll-off of phase locking [27]. Subcortical synchrony appears necessary and sufficient to generate the response [28], and this holds across the frequency range encompassing the stimulus F0 used here [29], within which a cortical contribution has nonetheless been documented [13,15]. The responses reported here are therefore predominantly subcortical in origin, with cortical and corticofugal contributions that cannot be excluded and were not measured. Accordingly, the differences described below are referred to as changes in speech-evoked neural encoding reflected in the FFR: the present data localize neither the response nor its modulation to a single generator within this pathway.

### 4.2. Specificity of the Observed Pattern: Sustained Metrics, Onset Timing, and Interaural Differences

The dissociation between sustained and onset measures is informative rather than incidental. Peak latencies index the timing of neural conduction and the synchrony of onset responses, whereas sustained pitch-tracking metrics index the precision with which the periodicity of the stimulus is represented across the steady-state portion of the response. These are separable properties: a change in the fidelity of periodicity encoding does not entail a shift in conduction timing, and stable onset latencies are therefore what would be expected if the difference between recordings reflects encoding precision rather than altered transmission along the auditory pathway. The stability of these onset measures also serves an evidential function in a single-case design. Global changes in recording conditions, electrode impedance, arousal, or signal quality would be expected to affect onset timing, signal-to-noise ratio, and sustained metrics together. The observation that onset latencies, SNR, and onset cross-correlation all remained comparable while both sustained pitch metrics differed with moderate to large effect sizes argues against a non-specific measurement artifact and constitutes the principal internal control available in this design.

Within this pattern, the latency data are themselves informative. Onset components (V, A) were stable across time points, as were onset cross-correlation and signal-to-noise ratio. The one consistent latency shift occurred at peak C, which does not index conduction along the auditory pathway but marks the transition from the onset response to the sustained periodic portion of the FFR; its earlier occurrence at the post-assessment in all six stimulus–ear combinations indicates that the periodic response was established earlier relative to stimulus onset. This falls on the same side of the dissociation as the pitch-tracking metrics rather than contradicting it: what differed between recordings concerns the sustained representation of stimulus periodicity, not the timing of transmission along the auditory pathway. Because latencies could be measured only on fully averaged waveforms, this observation is descriptive and was not tested statistically; it is reported because its consistency across all six combinations is notable and warrants examination in future work with sufficient observations for inferential analysis.

The difference between ears requires a more cautious reading. Effects were larger in the left ear (pitch strength *r* = 0.68; pitch error *r* = −0.52) than in the right (*r* = 0.46 and −0.33), and only the left-ear pitch strength comparison reached the conventional significance threshold. The stimulation applied was systemic and not lateralized, and there is no mechanistic basis on which a lateralized effect would be predicted. Two considerations argue against over-interpreting this asymmetry. First, the differences are in magnitude rather than direction: both ears changed in the same direction on both metrics, and the right-ear effect sizes remain moderate to large. Second, single-ear comparisons rest on nine observations per condition and are correspondingly underpowered, such that effects of this magnitude may fail to reach significance for reasons of sample size alone; the right-ear pitch strength comparison (*p* = 0.112) is consistent with this interpretation.

A genuine interaural difference in the responsiveness of auditory encoding is a plausible reading of this pattern. Functional lateralization of central auditory processing is well established, and asymmetry is not anomalous at this age: in a cohort of 37 typically developing children aged 8 to 10 years, ear was found to be a more consistent predictor of variability in speech-evoked responses than either age or sex [11], although those analyses concerned response latencies rather than pitch-tracking metrics. In the present case, the two ears were recorded within the same session, under identical acquisition conditions and with the same physiological state, so the interaural comparison is not subject to the between-session sources of variation that affect the pre–post comparison. What the present data cannot do is distinguish a genuine differential responsiveness from sampling variability at *n* = 9 per condition. The combined-ear analysis, in which both metrics differed with large effect sizes, provides the more interpretable summary of the observed pattern.

### 4.3. Potential Network Mechanisms and the Role of the Prefrontal Cortex

Any account of how peripherally applied microcurrent stimulation might relate to changes in speech-evoked neural encoding must be stated as a hypothesis, since no measurement in the present study bears on it directly. No cortical activity was recorded, no measure of autonomic function was obtained, and no comparison condition was available; the design therefore cannot establish that the stimulation altered prefrontal activity, nor that any such alteration mediated the observed differences. What follows is a description of the pathway that would have to be operative for such an effect to occur, offered so that it can be tested rather than assumed.

The proposed route involves three successive steps, each with a different evidential status. The first is peripheral-to-central transmission: stimulation is applied at distal surface sites, so any central consequence would have to be mediated by afferent input, plausibly through somatosensory and autonomic pathways converging on brainstem and diencephalic regulatory structures. That peripheral electrical stimulation can modulate central excitability through these pathways is established rather than speculative: it is a sufficiently consistent phenomenon to have been the subject of systematic review of the stimulus parameters required to elicit it [30], and stimulation delivered to a limb at sub-motor-threshold intensity has been shown to alter both cortical and subcortical components of the somatosensory evoked potential, indicating that afferent input from distal sites reaches and modifies central processing at more than one level [31]. What has not been demonstrated is that the specific electrode configuration, current range, and proprietary stimulation algorithm of the device used here engage these pathways, or that any such engagement would extend to the auditory system; this remains an assumption of the manufacturer’s rationale rather than a published finding.

The second is engagement of anterior regulatory networks: preliminary work using a comparable distal electrode topology, though with the directional electrode positioned at C7 rather than Fpz, has reported increased anterior alpha activity following superficial neurostimulation [19], which is consistent with, though far from establishing, modulation of frontal network state. The third is descending influence on auditory encoding: corticofugal projections from prefrontal and auditory cortical regions to the inferior colliculus and brainstem are well characterized and provide an established mechanism by which higher-order network state can modulate the precision of subcortical auditory representation [15,32]. Only this final step rests on a substantial independent literature; the first two do not.

The evidence base for superficial microcurrent neuromodulation in pediatric neurodevelopmental populations remains limited in ways that should be stated plainly. The available studies are few, generally small, and heterogeneous in outcome measure; several are open-label or lack sham control, and the internal modulation algorithm of the device is proprietary, which precludes independent characterization of the delivered waveform. Reported outcomes have centered on sleep, autonomic regulation, and behavioral measures [19,20,21,22] rather than on auditory or electrophysiological endpoints, so no prior work establishes an expected effect on speech-evoked neural encoding. The present observation should not be read as strengthening this evidence base: it is a single uncontrolled case in which no causal inference is available, and its contribution lies in demonstrating that FFR-derived measures are sensitive enough to be used as outcome measures in the controlled studies that would be required to test the hypothesis outlined above.

Independently of the clinical context of this observation, the prefrontal cortex (PFC) is well established as a high-level integrative hub involved in attentional control, temporal organization, and top-down modulation of acoustic information, particularly relevant in complex listening situations where auditory signals are ambiguous or degraded. Electrophysiological models of central auditory processing attribute to frontal generators a role in the preattentive detection of acoustic change and in the initiation of attentional switching toward behaviorally relevant sounds [16,33], mechanisms that plausibly bear on the precision with which incoming acoustic detail is encoded. Ventrolateral prefrontal regions have further been implicated in processing complex, communicatively relevant sounds, supporting abstract auditory object representation [34]. The PFC also supports auditory working memory and executive control, facilitating sequential integration and goal-directed processing; disruptions in these mechanisms may result in reduced temporal precision and unstable pitch encoding, as reflected in FFR measures [13,14]. Through corticofugal pathways, the PFC exerts top-down influence on subcortical auditory structures including the brainstem and inferior colliculus, providing a plausible—though here untested—mechanism by which distributed regulatory networks could, in principle, be relevant to sustained pitch-tracking measures such as those reported here [15,32].

### 4.4. Clinical Relevance of FFR as a Monitoring Biomarker in FASD

Fetal alcohol spectrum disorder (FASD) is characteristically associated with alterations in fronto-subcortical networks affecting executive functions, attention, and behavioral regulation. Within this framework, central auditory processing difficulties in children with FASD may be better understood as the functional expression of disrupted top-down control mechanisms rather than as a primary auditory deficit [35,36].

The findings of the present case are consistent with the potential utility of the FFR as a candidate objective measure for detecting within-subject change in auditory encoding in pediatric populations with FASD, particularly when behavioral assessments are limited or unreliable. The sensitivity of the FFR to neurodevelopmental variability has been demonstrated in pediatric cohorts, including normative datasets in newborns and infants, supporting its utility as a biomarker of early auditory system integrity and temporal precision [37]. Beyond FASD, this feasibility observation is consistent with a broader clinical need across pediatric neurodevelopmental and complex-diagnosis populations for objective, non-behavioral physiological monitoring tools that do not depend on a child’s ability to cooperate with structured behavioral testing.

Framed in these terms, the contribution of this observation is best understood as a proof of concept for the measurement approach rather than as a clinical report of an individual patient. What is being demonstrated is that speech-evoked FFRs can be acquired, quantified, and compared across time points in a child with FASD with sufficient stability to resolve change in sustained pitch-encoding metrics while onset timing and recording-quality indices remain unchanged.

A single participant is not incidental to this aim. Restricting the observation to one child holds constant the anatomical, maturational, and audiological sources of variance that would otherwise dominate a between-subject comparison, so that the question of whether the measurement is sensitive enough to detect within-subject change can be addressed on stable and standardized data.

The scope of what such a demonstration establishes is correspondingly narrow: it concerns the feasibility and sensitivity of the method, not the efficacy of the intervention, the validity of the FFR as a diagnostic index, or the generalizability of the pattern observed. These questions require the controlled designs outlined below, for which the present work is intended to provide the methodological groundwork.

### 4.5. Limitations

This study describes a single clinical case without a control or sham condition, and without parallel behavioral outcome measures. Behavioral assessment is frequently unreliable in children with FASD and other neurodevelopmental conditions, which is precisely why this study relied on objective, non-behavioral measures. Block-level comparisons rest on repeated acquisitions from a single participant, with the interpretive constraints set out in Section 2.7.

Several specific sources of uncontrolled variation warrant explicit consideration. First, the approximately twelve-week interval between assessments coincided with the transition from the academic year to the school holiday period. Although this ensured the absence of concurrent formal therapies or school-based support, it also entailed a substantial change in the participant’s daily environment, including the cessation of sustained academic and attentional demands. Such contextual changes may plausibly influence arousal, fatigue, and attentional state, all of which can modulate speech-evoked neural responses, and cannot be disentangled from the intervention period in the present design.

Second, normal maturational change over this interval cannot be formally excluded, although several considerations bear on its plausibility here. Subcortical auditory maturation continues throughout the first decade, but normative data indicate that the sustained portion of the speech-evoked response is comparatively stable by this age: in a cohort of 37 typically developing children aged 8 to 10 years, absolute latencies of the sustained components differed little across the three age bands, and this stability was interpreted as indicating that encoding of the temporal envelope and fine structure of speech is largely established by this developmental stage [11]. The participant described here was eight years old at both assessments, approximately twelve weeks apart. Maturational change of the magnitude required to account for the observed differences over that window would therefore be atypical for this age range, although it cannot be ruled out in a single case, and no test–retest reliability data were obtained for this participant to establish the expected magnitude of spontaneous variation in these metrics. Test–retest consistency of speech-evoked responses has been characterized in typically developing children [38], but those values were not established for this participant under the present protocol. Third, repeated exposure to the recording procedure itself may have contributed to greater tolerance of the protocol at the second assessment. For these reasons, the observed differences should be interpreted as within-subject change occurring over a defined observation period, and not as an effect attributable to the concurrent neuromodulation intervention.

Additional methodological limitations should be noted. No standardized quantitative behavioral or functional outcome measures were collected during the observation period; the family reported unquantified impressions of change in everyday functioning, but these observations were neither structured nor blinded and cannot be related to the electrophysiological findings. Accordingly, no inference is drawn here that the observed FFR differences correspond to clinically meaningful functional improvement. Furthermore, although recording conditions were standardized and acquisition was initiated only after EEG stabilization at both time points, arousal state was not quantified using a formal staging criterion, and subtle variation in state during acquisition cannot be fully excluded. Finally, waveform components were identified by a single experienced examiner, and no inter-rater reliability assessment was performed; although peak identification followed standardized criteria, the absence of independent verification is acknowledged.

The proprietary nature of the neuromodulation device’s internal algorithm further limits mechanistic interpretation. Findings should be confirmed in larger, controlled studies integrating electrophysiological and behavioral outcomes.

### 4.6. Clinical and Research Implications

Despite its exploratory nature, this case report suggests that FFRs may serve as an objective electrophysiological measure for monitoring within-subject change in neural speech encoding in pediatric FASD. Future studies should employ controlled designs, include behavioral correlates, and examine cortical oscillatory dynamics to clarify the mechanisms and monitoring value of FFR-based biomarkers in neurodevelopmental populations, and, separately, to formally test any therapeutic hypothesis regarding non-invasive neuromodulation using appropriately controlled designs. Establishing whether FFR-derived change corresponds to functional change will require controlled designs incorporating validated behavioral instruments alongside electrophysiological measures.

## 5. Conclusions

This proof of concept case study documents measurable within-subject change in sustained FFR-derived pitch encoding across two assessment time points in a pediatric patient with FASD during non-invasive neuromodulation. Increased pitch strength and reduced pitch error were observed while onset response timing, signal-to-noise ratio, and onset stimulus–response correlation remained stable; the one consistent latency shift occurred at the transition between the onset and sustained portions of the response, on the same side of this dissociation as the pitch-tracking metrics. These observations support the technical feasibility of repeated FFR assessment in this population and indicate that FFR-derived pitch metrics are sensitive enough to register within-subject variation over an interval of approximately twelve weeks. Because this is a single uncontrolled case, the findings establish neither a diagnostic role for the FFR nor any effect of the concurrent neuromodulation intervention. They should be read as hypothesis-generating, and require replication in larger, controlled cohorts before the monitoring utility of FFR-based measures in FASD can be established.

## Figures and Tables

**Table 1:** Block-level statistical comparisons of sustained and onset FFR metrics between pre- and post-assessment (Mann–Whitney U tests).

Variable	Ear	Median Pre	Median Post	*U*	*p*	*r*
Pitch strength	Both	0.39	0.71	67.00	0.003 **	0.59
	RE	0.42	0.72	22.00	0.112	0.46
	LE	0.35	0.71	13.00	0.017 *	0.68
Pitch error	Both	7.25	2.54	87.50	0.019 *	−0.46
	RE	6.80	2.72	27.00	0.251	−0.33
	LE	7.70	1.23	19.50	0.070 ^†^	−0.52
SNR	Both	0.51	0.53	146.00	0.624	−0.10
	RE	0.51	0.53	38.50	0.894	0.05
	LE	0.50	0.53	30.00	0.377	−0.26
Cross-correlation (3.98–9 ms)	Both	0.03	0.06	152.00	0.764	−0.06
	RE	0.03	0.06	40.50	1.000	0.00
	LE	0.00	0.07	35.00	0.659	−0.14

* *p* < 0.05; ** *p* < 0.01; ^†^ *p* < 0.10. *U* = Mann–Whitney U statistic (smaller of the two rank sums); r = rank-biserial correlation, with positive values indicating higher values at the post-assessment and negative values indicating lower values. RE = right ear; LE = left ear; Both = combined-ear analysis (*n* = 18 per condition); single-ear analyses (*n* = 9 per condition). All tests are two-tailed. Reported p-values are uncorrected for multiple comparisons (see Section 2.7).

## Data Availability

The data presented in this case report are available from the corresponding author on reasonable request, subject to the confidentiality and privacy restrictions applicable to identifiable pediatric clinical data.

## Supplementary Materials

The following supporting information can be downloaded at: https://www.mdpi.com/article/10.3390/children13081116/s1. Table S1: Frequency-following response (FFR) peak latencies for /da/, /ba/, and /ga/ speech stimuli at the two assessment time points; Table S2: Stimulus-specific pitch-tracking correlation values for /da/, /ba/, and /ga/ speech stimuli at the two assessment time points; Figure S1: Temporal dynamics of pitch tracking for the syllable /ba/ across the two assessment time points; Figure S2: Time–frequency representation of the frequency-following response using running FFT analysis.

## Abbreviations

The following abbreviations are used in this manuscript:

FASD	Fetal Alcohol Spectrum Disorder
FFR	Frequency-Following Response
APD	Auditory Processing Disorder
SNR	Signal-to-Noise Ratio
tDCS	Transcranial Direct Current Stimulation
PFC	Prefrontal Cortex
SPL	Sound Pressure Level
F_0_	Fundamental Frequency
