# Supplementary material for: Frequency-Following Responses for Objective Neurophysiological Monitoring in Pediatric Fetal Alcohol Spectrum Disorder: A Proof of Concept Case Study During Non-Invasive Neuromodulation
Source: Children (Basel). 2026 Aug 20;13(8):1116. doi: 10.3390/children13081116 (PMC13511160; doi:10.3390/children13081116)
Supplement: Supplementary file 1 [file children-13-01116-s001.zip › children-4464026-supplementary.pdf]

children

MDPI

Supplementary Materials

Frequency-Following Responses for Objective Neurophysiological Monitoring in Pediatric Fetal Alcohol Spectrum Disorder: An Exploratory Case Study During Non-Invasive Neuromodulation.

S1. Supplementary Methods

Detailed FFR acquisition, signal processing, spectral/pitch-tracking analysis, and the non-invasive neuromodulation protocol are reported in full in the main manuscript (Sections 2.4–2.6) and are not duplicated here. The broader audiological and neurophysiological assessment battery used to characterize the participant's auditory processing profile prior to the FFR-based observation is described below, as it falls outside the scope of the main-text Methods.

S1.1. Audiological and Neurophysiological Assessment Protocol

A comprehensive audiological evaluation was conducted to exclude peripheral hearing impairment and to characterize auditory function across peripheral, subcortical, and cortical levels. Pure-tone audiometry by air conduction was performed, including high-frequency and ultra-high-frequency thresholds. Tympanometry and ipsilateral and contralateral acoustic reflexes were obtained to assess middle ear function and brainstem reflex pathways. Cochlear function was evaluated using transient-evoked otoacoustic emissions (TEOAEs) and distortion product otoacoustic emissions (DPOAEs). Otoacoustic emissions were recorded both in baseline conditions and during ipsilateral and contralateral acoustic stimulation to assess efferent auditory system function through suppression paradigms.

S1.2. Auditory Processing Assessment

Functional auditory processing was assessed using a battery of behavioral and electrophysiological measures. Masking-Level Differences (MLD) were obtained to evaluate binaural interaction at the brainstem level. Based on the overall pattern of results, the clinical profile was consistent with an auditory processing disorder (APD), characterized primarily by deficits in auditory attention, auditory memory, and speech perception in noise.

S1.3. Broader Electrophysiological Assessment

Auditory brainstem responses (ABR) were recorded to assess neural conduction along the auditory brainstem pathway. In addition to the frequency-following responses (FFR) reported in the main manuscript, cortical auditory evoked potentials (CAEPs) were recorded to assess higher-order auditory processing. The CAEP battery included the P1–N1–P2 complex obtained at both conventional and high stimulation rates, as well as cognitive auditory potentials including P300 and mismatch negativity (MMN), providing information on auditory discrimination, attention, and sensory memory mechanisms.

S1.4. Diagnostic Integration

The integration of behavioral, audiological, and electrophysiological findings supported a diagnosis of auditory processing disorder in the context of Fetal Alcohol Spectrum Disorder (FASD). The observed auditory deficits were interpreted as reflecting dysfunction within fronto-subcortical regulatory networks rather than primary peripheral auditory pathology.

 https://doi.org/10.3390/children13081116

S2. Supplementary Results

S2.1. Peak Latencies

Peak latencies corresponding to the V–O complex (V, A, C, D, E, F, O) for each stimulus (/da/, /ba/, /ga/) and ear at the two assessment time points are reported in Supplementary Table S1. Peaks were identified on the waveform obtained by averaging all three recording blocks per stimulus and ear (3072 sweeps), using an 80-ms display window to improve visualization of response transitions; block-level peak identification was not attempted, as the signal-to-noise ratio of individual blocks did not permit reliable marking of individual components. Latency values are therefore presented descriptively and were not subjected to inferential statistical testing. Identical identification criteria were applied at both time points.

S2.2. Stimulus-Specific Pitch-Tracking Correlation Values

Pitch-tracking correlation values for each stimulus (/da/, /ba/, /ga/) and ear are provided in Supplementary Table S2. These values correspond to the stimulus–response correlation derived from the sustained portion of the FFR, computed on the waveform obtained by averaging all three recording blocks per stimulus and ear. They are presented descriptively and were not subjected to inferential statistical testing; the onset stimulus–response cross-correlation reported in Table 1 of the main manuscript is a separate measure, computed over the 3.98–9 ms window.

S2.3. Temporal Dynamics of Pitch Tracking

Temporal pitch-tracking trajectories derived from the Brainstem Toolbox are illustrated for the syllable /ba/ in Supplementary Figure S1. These plots show the evolution of the extracted fundamental frequency estimate across the response window at the two assessment time points. These representations are provided for descriptive visualization and were not subjected to separate statistical testing.

S2.4. Time–Frequency Representations (Running FFT)

Time–frequency representations of the FFR obtained using running fast Fourier transform (FFT) analysis are shown in Supplementary Figure S2. These plots illustrate the temporal distribution of spectral energy within the analyzed response window at the two assessment time points. FFT representations are included for completeness and were not analyzed inferentially.

https://doi.org/10.3390/children13081116

S3. Supplementary Figures and Tables

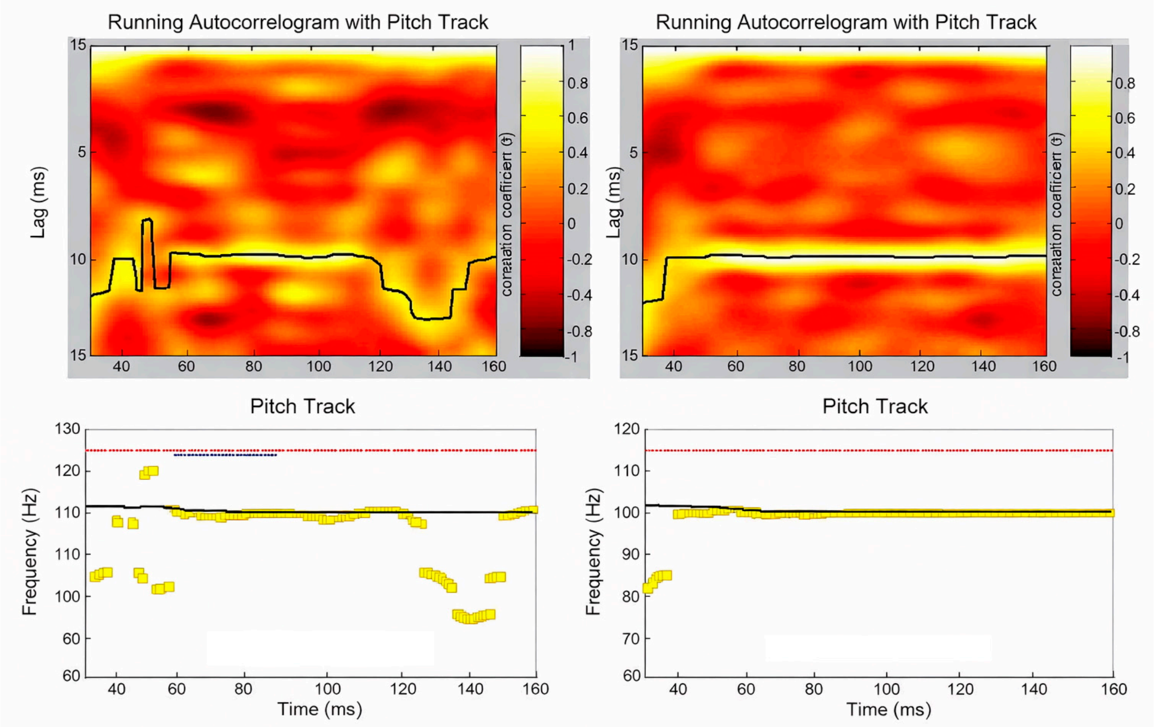

Supplementary Figure S1. Temporal dynamics of pitch tracking for the syllable /ba/ across the two assessment time points. Running autocorrelograms with superimposed pitch tracks (top panels) and corresponding pitch-tracking trajectories (bottom panels) illustrate the temporal evolution of fundamental frequency (F0) encoding for the syllable /ba/. Pre-assessment recordings are shown in the left column and post-assessment recordings in the right column.

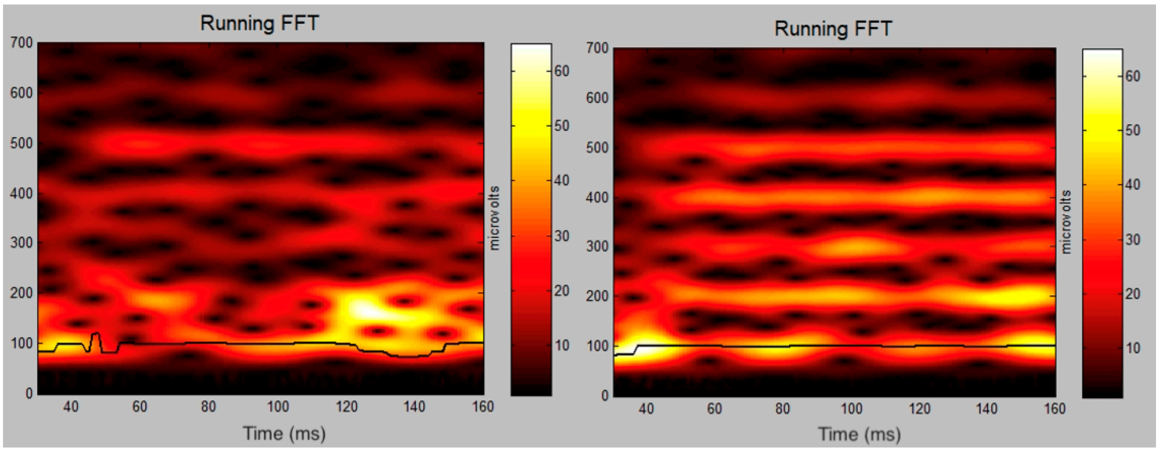

Supplementary Figure S2. Time–frequency representation of the frequency-following response using running FFT analysis. Running fast Fourier transform (FFT) representations of the FFR illustrate the temporal evolution of spectral content across the response window for the syllable /ba/. The horizontal axis represents time (ms) and the vertical axis represents frequency (Hz); color intensity reflects response amplitude (μV). The superimposed line indicates the extracted pitch-tracking trajectory corresponding to F0. Panels display the pre-assessment (left) and post-assessment (right) recordings for direct visual comparison. These representations are provided for descriptive visualization only and were not subjected to statistical analysis.

https://doi.org/10.3390/children13081116

Supplementary Table S1. Frequency-following response (FFR) peak latencies for /da/, /ba/, and /ga/ speech stimuli at the two assessment time points.

Stimulus	Ear	Time point	V	A	C	D	E	F	O
/da/	Left	Pre	7.42	9.75	20.55	25.28	34.72	42.90	52.58
/da/	Left	Post	7.65	10.05	15.90	24.79	34.05	42.79	52.95
/da/	Right	Pre	6.98	9.60	19.95	24.08	34.05	44.18	56.10
/da/	Right	Post	7.50	9.45	14.92	24.90	34.28	43.42	53.18
/ba/	Left	Pre	7.58	8.78	21.45	27.45	33.45	44.92	58.28
/ba/	Left	Post	7.50	10.05	15.22	21.52	36.98	43.28	53.18
/ba/	Right	Pre	7.20	9.30	21.22	24.08	35.25	47.70	54.08
/ba/	Right	Post	7.65	10.65	15.52	25.20	37.50	45.22	52.95
/ga/	Left	Pre	7.65	10.58	20.10	24.60	34.65	44.78	58.80
/ga/	Left	Post	7.80	9.45	15.52	25.20	34.05	44.25	48.45
/ga/	Right	Pre	6.98	9.52	19.58	23.62	33.38	42.68	54.45
/ga/	Right	Post	7.65	10.88	15.52	24.08	34.12	43.12	48.98

Peak latencies are reported in milliseconds (ms) for peaks V, A, C, D, E, F, and O, separately for the left and right ears. Peaks V and A correspond to the onset portion of the FFR, peak C marks the transition between the onset response and the sustained periodic portion, and peaks D–E–F–O correspond to the sustained portion of the response.

Supplementary Table S2. Stimulus-specific pitch-tracking correlation values for /da/, /ba/, and /ga/ speech stimuli at the two assessment time points.

Stimulus	Ear	Pitch tracking correlation (Pre)	Pitch tracking correlation (Post)
/da/	Right	0.85	0.83
/da/	Left	0.76	0.85
/ba/	Right	0.66	0.76
/ba/	Left	0.51	0.86
/ga/	Right	0.61	0.83
/ga/	Left	0.72	0.69

Pitch-tracking correlation values derived from FFR recordings are presented separately for right and left ears at the two assessment time points. Values reflect the degree of temporal alignment between the stimulus fundamental frequency contour and the neural response. Values were computed on the waveform obtained by averaging all three recording blocks per stimulus and ear.

Disclaimer/Publisher's Note: The statements, opinions and data contained in all publications are solely those of the individual author(s) and contributor(s) and not of MDPI and/or the editor(s). MDPI and/or the editor(s) disclaim responsibility for any injury to people or property resulting from any ideas, methods, instructions or products referred to in the content.

https://doi.org/10.3390/children13081116
